# Supplementary material for: Mouse Models of Human Pathogenic Variants of TBC1D24 Associated with Non-Syndromic Deafness DFNB86 and DFNA65 and Syndromes Involving Deafness
Source: Genes (Basel). 2020 Sep 24;11(10):1122. doi: 10.3390/genes11101122 (PMC7598720; doi:10.3390/genes11101122)
Supplement: Supplementary file 1 [file genes-11-01122-s001.pdf]

Figure S1

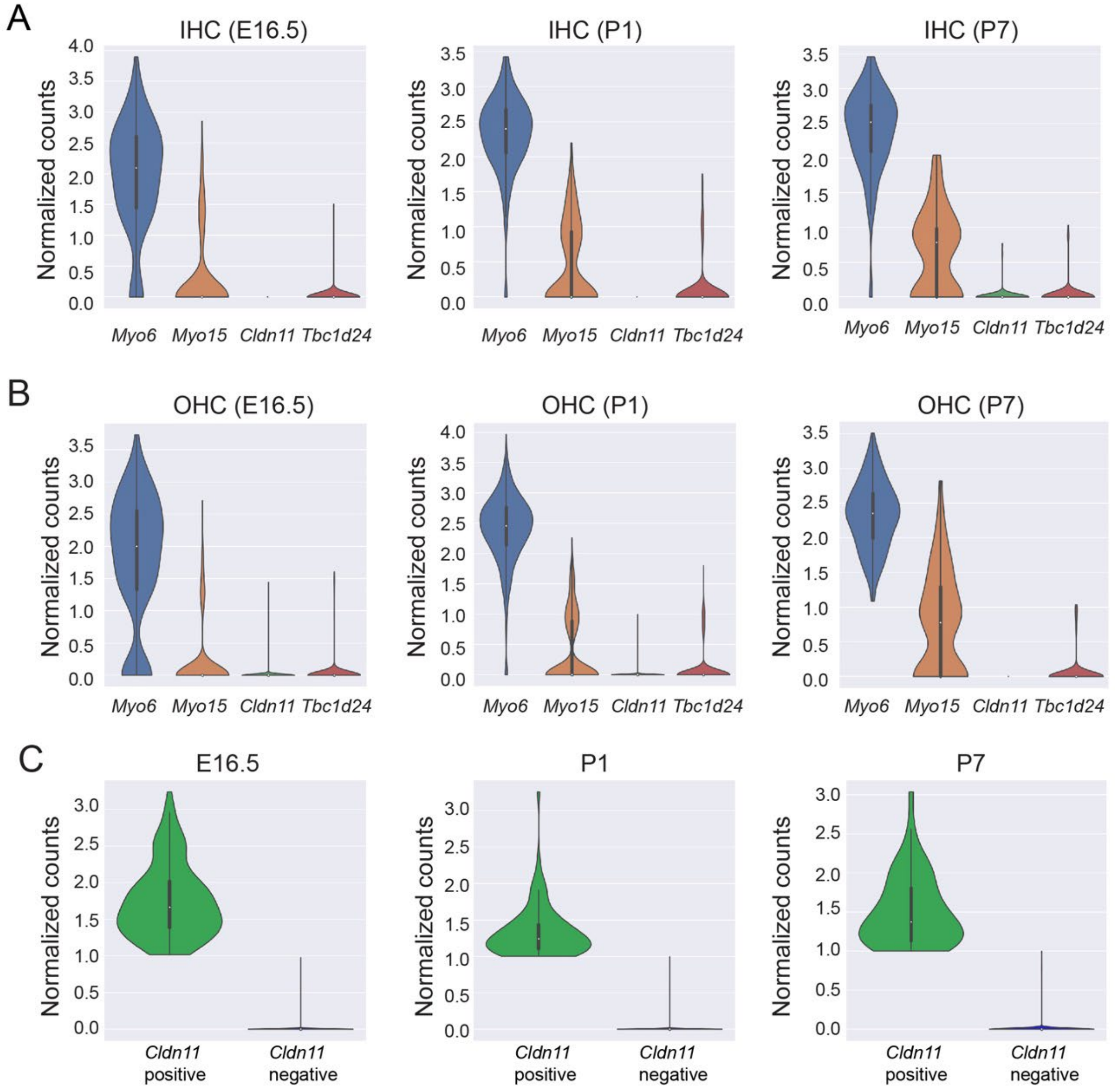

Supplementary Figure 1. Single cell RNA-sequencing of inner and outer hair cells. Single cell RNA-sequencing of developing inner and outer hair cells reported by Kolla and colleagues [1] demonstrates that *Tbc1d24* expression in hair cells is at background levels. Each violin plot demonstrates gene expression in normalized counts of *Myo6* and *Myo15*, genes known to be expressed by hair cells, and *Cldn11* that is known to be expressed in basal cells of stria vascularis, but not in hair cells. *Cldn11* displays either not detectable or a background levels in hair cells, similar to *Tbc1d24*. (A) Violin plots showing expression levels of *Myo6*, *Myo15*, *Cldn11*, and *Tbc1d24* in inner hair cells at E16.5, P1 and P7. (B) Violin plots showing expression levels of *Myo6*, *Myo15*, *Cldn11*, and *Tbc1d24* in outer hair cells at E16.5, P1 and P7. (C) Violin plots showing expression levels of *Cldn11* in *Cldn11*-positive and *Cldn11*-negative cells at E16.5, P1 and P7.

Figure S2

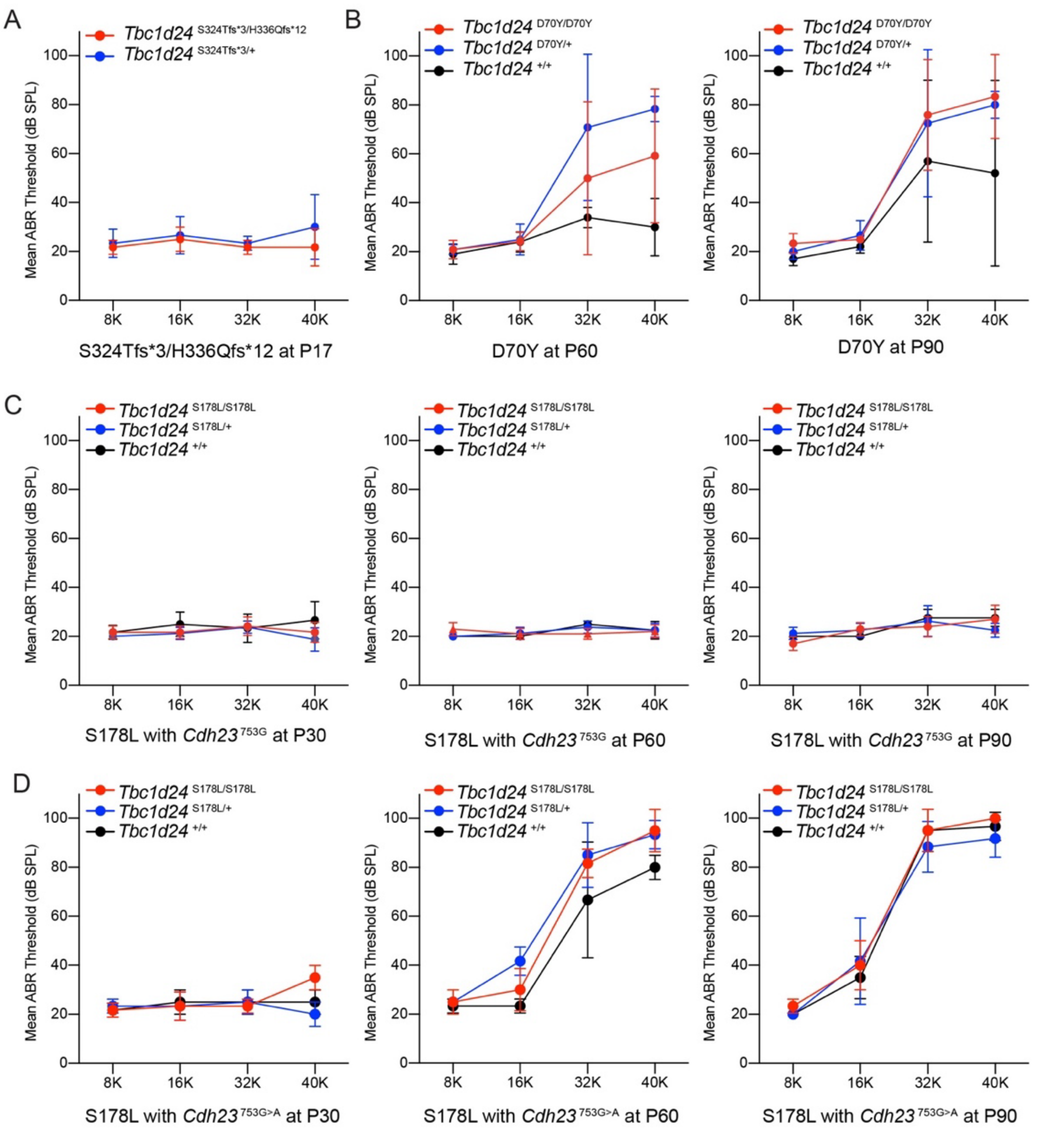

Supplementary Figure 2. ABR thresholds of *Tbc1d24* wild type and mutant mice. (A) Mean ABR thresholds of *Tbc1d24* compound heterozygous p.Ser324Thrfs*3 (S324Tfs*3)/p.His336Glnfs*12 (H336Qfs*12) (n=3) and heterozygous p.Ser324Thrfs*3 mice (n=3) show normal hearing at P17. Because compound heterozygous p.Ser324Thrfs*3/p.His336Glnfs*12 mice usually die between the ages of P15 and P20, only ABR thresholds at P17 are shown. (B) Mean ABR thresholds of *Tbc1d24* homozygous p.Asp70Tyr (D70Y) (n=6), heterozygous p.Asp70Tyr (n=5) and wild type littermates (n=5) at P60 and P90, related to figure 4A. (C) Mean ABR thresholds of *Tbc1d24* homozygous p.Ser178Leu (S178L) with *Cdh23* wild type (*Cdh23*^{753G}) (n=6), heterozygous p.Ser178Leu with *Cdh23*^{753G} (n=4) and wild type littermate (n=3) mice at P30, P60 and P90, related to figure 4C. (D) Mean ABR thresholds of *Tbc1d24* homozygous p.Ser178Leu with *Cdh23* c.753G>A (*Cdh23*$^{753G>A}$) (n=3), heterozygous p.Ser178Leu with *Cdh23*$^{753G>A}$ (n=3) and wild type littermates (n=3) at P30, P60 and P90, related to figure 4C. Although mice with this allele showed age-related high frequency hearing loss, no significant difference was detected between the p.Ser178Leu mutant mice and their wild type littermates. All data represent mean ± SD.

Figure S3

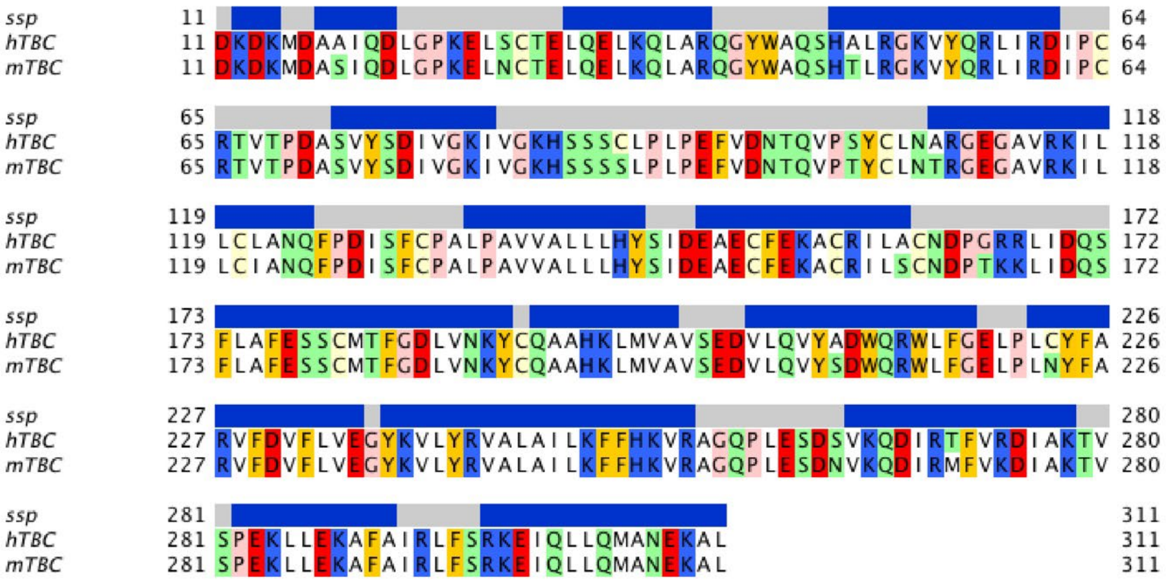

Supplementary Figure 3. Sequence alignment of human and mouse TBC domain of TBC1D24 protein obtained using ClustalW (https://www.ebi.ac.uk/Tools/msa/clustalo/). The predicted secondary elements are indicated at the top of the sequence as blue (helix) and gray (coil) bars.

Figure S4

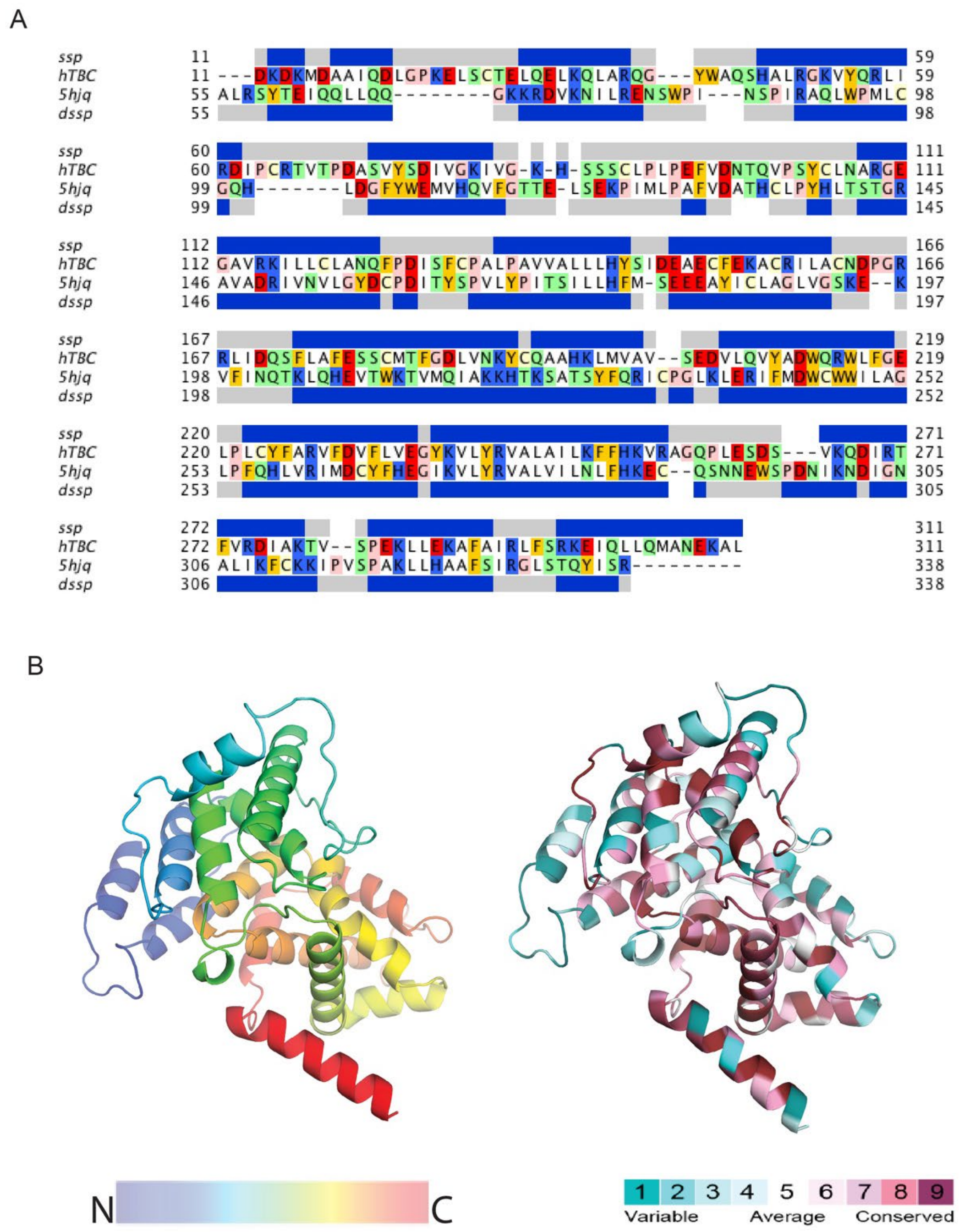

Supplementary Figure 4. Structural model of the human TBC (hTBC) domain of human TBC1D24 (hTBC1D24). (A) Sequence alignment used during the modeling procedure of the TBC domain corresponded to human TBC1D24 (residues 11-311) and *Drosophila melanogaster* Skywalker hTBC1D24 (residues 55-338). The secondary structural elements for the template were assigned with DSSP [2] (dssp), as well as those predicted with Psipred (ssp), which are shown as blue (helix) and gray (coil) bars below and above the corresponding sequences. The model obtained (B) is shown as a cartoon (blue at the N-terminal towards red at the C-terminal) on the left and by conservation scores on the right.

Figure S5

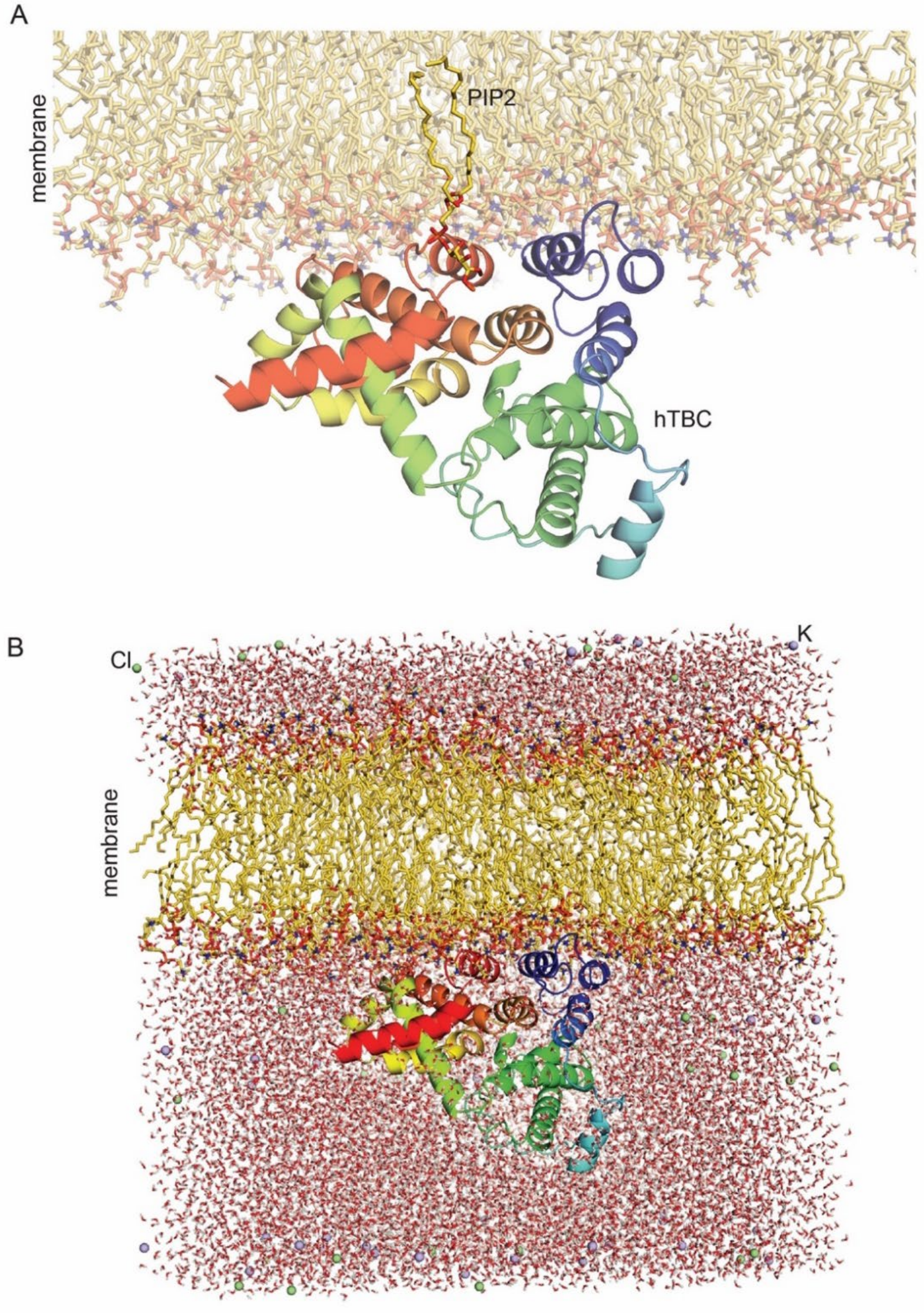

Supplementary Figure 5. Representation of the Molecular Dynamics setup. (A) TBC domain of hTBC1D24 is bound to a PIP2 lipid in the presence of a membrane. Lipids are shown as sticks and the TBC domain as a cartoon. (B) The simulation box used during MD simulations where water molecules and lipids are shown as sticks, chloride and potassium ions as green and purple spheres and the TBC domain as a cartoon. The aliphatic chain of the lipids is yellow colored, while the heteroatoms are colored as follows: oxygen: red, phosphorus: orange, nitrogen: blue. The hydrogen atoms are white colored. In all setups, the x, y plane of the simulation box is parallel to the membrane average plane, while z is the perpendicular direction.

RMSD (Å)	hTBC (sim 1)	hTBC (sim 2)	mTBC (sim 1)	mTBC (sim 2)
**hTBC (ave, sim 1)**	1.8	3.5	4.2	3.7
**hTBC (ave, sim 2)**	3.6	1.6	3.2	4.0
**mTBC (ave, sim 1)**	4.4	3.3	1.4	4.3
**mTBC (ave, sim 2)**	3.7	4.0	4.1	1.8

Table S1. Observed difference in the structures of hTBC and mTBC. The table reports the average root mean square deviations (RMSDs) calculated on the protein backbone of the average structure of each simulation superimposed to either the whole sets of snapshots of the same simulation (grey shading) or the other simulations. Snapshots were taken every 1 ps.

ΔG (kcal/mol)	hTBC	mTBC	Ala-Ser-Ala
**Set 1**	-14.70 ± 0.11	-18.34 ± 0.15	-15.28 ± 0.07
**Set 2**	-14.96 ± 0.10	-18.45 ± 0.19	-15.32 ± 0.05
**Average**	-14.83 ± 0.10	-18.40 ± 0.18	-15.30 ± 0.06
**ΔΔG**	0.47 ± 0.17	-3.10 ± 0.24	

Table S2. Free energy differences (ΔG) from the FEP simulations. Values refer to the alchemic transformation from Ser178 to leucine. Two independent calculations were carried out for each system (Sets 1 and 2). The ΔΔG is calculated as the difference between the average ΔG of each system and that of the reference peptide.

1. Kolla, L.; Kelly, M.C.; Mann, Z.F.; Anaya-Rocha, A.; Ellis, K.; Lemons, A.; Palermo, A.T.; So, K.S.; Mays, J.C.; Orvis, J., et al. Characterization of the development of the mouse cochlear epithelium at the single cell level. *Nat Commun* **2020**, *11*, 2389, doi:10.1038/s41467-020-16113-y.
2. Kabsch, W.; Sander, C. Dictionary of protein secondary structure: pattern recognition of hydrogen-bonded and geometrical features. *Biopolymers* **1983**, *22*, 2577-2637, doi:10.1002/bip.360221211.
